# Natural and synthetic nanovectors for cancer therapy

**DOI:** 10.7150/ntno.77564

**Published:** 2023-03-05

**Authors:** Aziz Eftekhari, Carola Kryschi, David Pamies, Sukru Gulec, Elham Ahmadian, Dawid Janas, Soodabeh Davaran, Rovshan Khalilov

**Affiliations:** 1 Department of Biochemistry, Faculty of Science, Ege University, Izmir 35040, Turkey; 2 Institute of Molecular Biology & Biotechnologies, Ministry of Science and Education Republic of Azerbaijan, 11 Izzat Nabiyev, AZ1073 Baku, Azerbaijan; 3 Department of Chemistry and Pharmacy, Physical Chemistry I and ICMM, Friedrich-Alexander University of Erlangen─Nuremberg, Egerlandstrasse 3, D-91058 Erlangen, Germany; 4 Department of Biomedical Sciences, University of Lausanne, Rue du Bugnon 7, 1005 Lausanne, Switzerland; 5 Swiss Centre for Applied Human Toxicology (SCAHT), University of Basel, Missionsstrasse 64, 4055 Basel, Switzerland; 6 Molecular Nutrition and Human Physiology Laboratory, Department of Food Engineering, İzmir Institute of Technology, 35430 Urla, İzmir, Turkey; 7 Kidney Research Center, Tabriz University of Medical Sciences, Tabriz, Iran; 8 Department of Organic Chemistry, Bioorganic Chemistry and Biotechnology, Silesian University of Technology, B. Krzywoustego 4, 44-100, Gliwice, Poland; 9 Department of Pharmaceutical Toxicology, Yeditepe University Faculty of Pharmacy, Istanbul, Turkey; 10 Department of Medicinal Chemistry, Faculty of Pharmacy, Tabriz University of Medical Sciences, Tabriz, Iran; 11 Department of Biophysics and Biochemistry, Baku State University, Baku, Azerbaijan; 12 Institute of Radiation Problems, National Academy of Sciences of Azerbaijan, Baku, Azerbaijan

**Keywords:** Nanoparticles, nanovectors, cancer, drug delivery, nanomaterials

## Abstract

Nanomaterials have been extensively studied in cancer therapy as vectors that may improve drug delivery. Such vectors not only bring numerous advantages such as stability, biocompatibility, and cellular uptake but have also been shown to overcome some cancer-related resistances. Nanocarrier can deliver the drug more precisely to the specific organ while improving its pharmacokinetics, thereby avoiding secondary adverse effects on the not target tissue. Between these nanovectors, diverse material types can be discerned, such as liposomes, dendrimers, carbon nanostructures, nanoparticles, nanowires, etc., each of which offers different opportunities for cancer therapy. In this review, a broad spectrum of nanovectors is analyzed for application in multimodal cancer therapy and diagnostics in terms of mode of action and pharmacokinetics. Advantages and inconveniences of promising nanovectors, including gold nanostructures, SPIONs, semiconducting quantum dots, various nanostructures, phospholipid-based liposomes, dendrimers, polymeric micelles, extracellular and exome vesicles are summarized. The article is concluded with a future outlook on this promising field.

## Introduction

The tremendous growth in the field of nanotechnology has dramatically impacted biomedicine and drug delivery. For example, the ability to target a payload of active pharmaceutical ingredients (API) to a specific location at a proper concentration and time has driven the development of new cancer treatments 1 with the use of so-called nanocarriers or nanovectors. Such units, with size on the order of nanometers, combined with polymeric matrices, offer a more controlled release profile, which can be beneficial for the treatment 2. In particular, nanocarrier contrast agents that assist cancer diagnosis has led to breakthrough advances in cancer treatment. In this context, albumin-conjugated paclitaxel or a liposomal form of doxorubicin are among the first nano-therapeutics with great potential for cancer patients, whose performance is much improved because of nanoscopic size 3, 4.

There is plenty of motivation to develop new treatment approaches because the current methods have serious drawbacks. The use of conventional chemotherapeutic agents requires high drug doses and induces different adverse effects, which affect patients considerably, as such treatments lack precise means of targeting the cancerous cells. Nanotechnology-based drug delivery systems have opened new avenues in this context by minimizing these limitations 5. For example, nanoformulations increase the elimination half-life of the drugs and preserve them in circulation for a longer time 6. Furthermore, drugs can be better guided to cancer cells with nanomaterials 7. In addition, nanocarriers impede the degradation of medications, diminish their renal clearance, and increase the benefits of cytotoxic chemicals 1. These reasons justify why nanocarriers as intelligent drug delivery vehicles have gained significant attention for controlled drug release and bio-imaging purposes. Furthermore, nanocarriers exploiting specific receptors for targeting are particularly hopeful 5. Such nanocarriers performing as nanovectors are commonly grafted with functional groups, or amphiphilic surfactants are used to encapsulate them to boost their performance, which could be otherwise hampered by issues with solubility in the aqueous media 8, 9.

The efficiency of a nanovector-based treatment depends on cellular uptake and retention time, known as enhanced permeability and retention (EPR). Thus, the researchers focus on nanovectors with increased EPR effects to have the highest impact on cancer cells. The cellular uptake of nanovectors occurs mainly by non-transporter-guided endocytosis mechanisms. Recent studies indicate that the directed permeation of nanovectors via membrane crossing should improve the outcome of cancer treatments. Nutrient transporters are more highly expressed in cancer cells than in noncancerous cells. Thus, transporter-mediated nanovector uptake strategies are beneficial for drug delivery. Moreover, the plasma membrane surface should also be considered during the design of nanovectors since the plasma membrane is selective and semipermeable for molecules. Thus, the characteristics of nanovectors, such as size, shape, and charge, are essential. The nanoparticle size plays a significant role in cellular uptake. As a rule of thumb, the nanoparticle size should be ~ 50 nm for an efficient rate of particle internalization. Regarding charge, it has been repeatedly noticed that charged surface area of the nanoparticles enhances cellular uptake. Lastly, the eucaryotic cells have a lipid bilayer membrane structure composed of a hydrophilic head and hydrophobic tail. Because the extracellular surface of the membrane is a crucial factor in cellular uptake, when nanovectors are designed, structural features of nanovectors should be considered to ensure appropriate cellular uptake.

**Figure 1:**
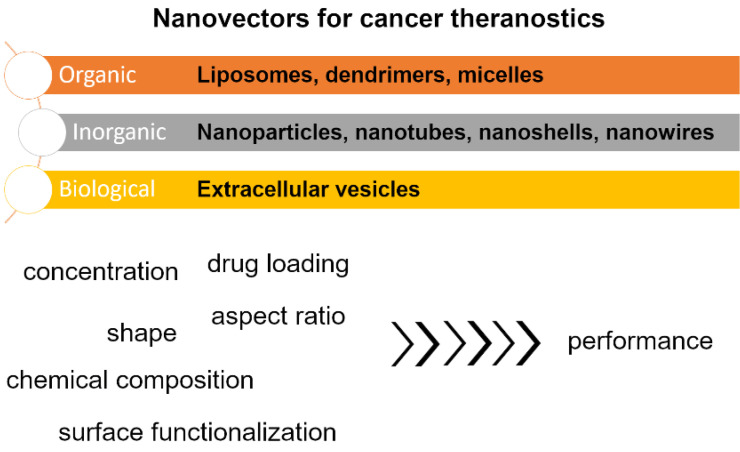
Overview of the scope of the article

This review article provides an overview of recently developed nanovectors for cancer therapies to highlight the merits of this new treatment approach. Examples of such formulations are thoroughly analyzed to gauge how their characteristics impact the performance (Figure 1) and what is their utility for drug delivery. The article is concluded with a future outlook, indicating the areas of this field that require the most attention nowadays.

## Organic nanovectors

### Liposomes

Liposomes are phospholipid-based spherical bilayer vesicles with a hydrophilic volume. They are among the most preferred nanocarriers because they can entrap hydrophilic and lipophilic drugs 10-12. A cell-like membrane shape, active group protection, low immunogenicity, safety, biocompatibility, and efficacy are among the various advantages of liposomes 13. The development of targeted liposomes by incorporating ligands such as folate, peptides, mannose, and transferrin enables the selective delivery of medications to a specific region 14, 15. As a matter of fact, the first FDA-approved nano-drug was Doxil in which doxorubicin was enclosed in a liposome.

However, liposomes elicit certain drawbacks, such as accelerated blood clearance (ABC) through the reticuloendothelial system (RES), a low entrapment rate, and a greater possibility for amphiphilic and hydrophilic drugs to escape from the liposomal vesicles, among others 16-18. In addition, their physicochemical properties affect the removal of liposomes by the mononuclear phagocyte system (MPS) and the RES.

**Figure 2:**
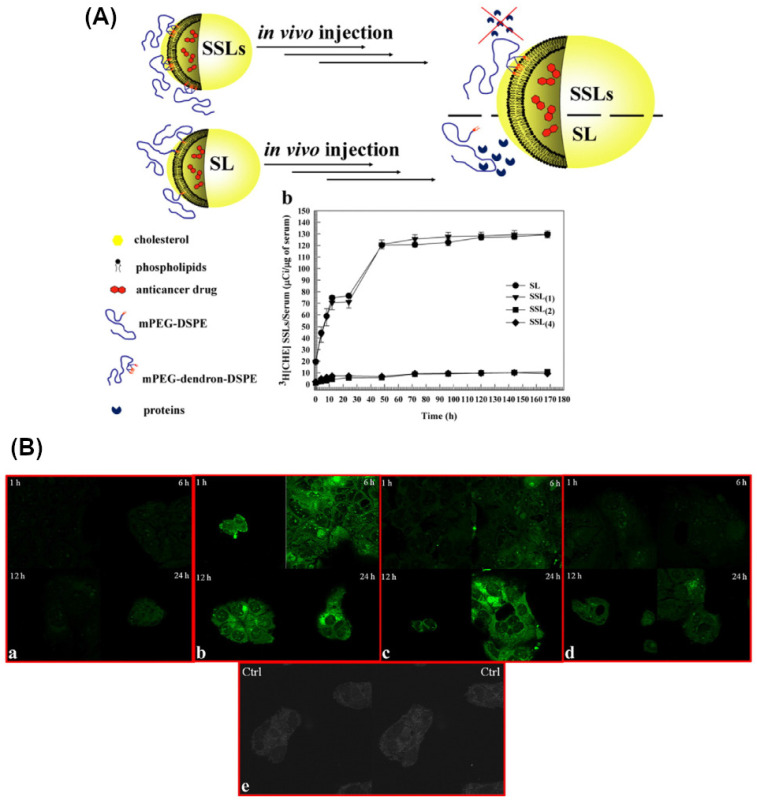
(A) Illustration of a super stealth liposome (SSL). The stability of the vesicle has been enhanced by attaching a single polyethylene glycol (PEG) chain to several phospholipids. When used as a drug delivery vehicle, this SSL exhibits a better biodistribution and antitumour effect than conventional stealth liposomes (SLs). The plot quantifies the stability of SSLs in serum. The leakage of ^3^[H]CHE from the SL and SSLs was used as a stability measurement. (B) Confocal laser scanning micrographs of CaCo-2 cells incubated with fluorescein-labelled SL and SSLs. CaCo-2 cells were treated with fluorescent SL or SSLs and incubated for different periods (1 h, 6 h, 12 h, and 24 h). a, SL. b, SSL_1_. c, SSL_2_. d, SSL_4_. e, Control (untreated CaCo-2 cells). Modified and reproduced with permission 19. Reproduced with permission. Copyright Elsevier (2015).

Pasut et al. 19 synthesized novel methoxy polyethylene glycol (PEG)-phospholipid structures that formed super stealth liposomes with a large anticancer agent payload (e.g., doxorubicin), a long half-life in the circulation, liposomal stability, and a good biodistribution profile. In addition, a cell culture study showed that super stealth liposomes have high intracellular uptake, acceptable toxicity, and improved therapeutic efficacy (low dose requirement of an anticancer agent) (Figure 2) 19. The promising performance was justified by using PEGylated nanoparticles which can increase drug accumulation at the desired site due to the reduction of macrophage clearance.

Moreover, the nanovectors can be made interactive. Stimulus-triggered drug delivery systems, which utilize the constituents of the tumour niche, have recently increased payloads delivered to cancerous tissue 20-23. For example, pH-stimulated liposomal formulations enhance drug release inside the tumour 24 due to different pH in the vicinity of the tumour. Paliwal et al. 25 developed hyaluronic acid-modified pH-stimulated liposomes that exhibited an increased affinity to CD44 receptor-overexpressing cells compared with normal cells.

Furthermore, liposomal systems can be used as theranostic apparatuses by simultaneously incorporating a diagnostic and therapeutic moiety 26. The second generation of liposomes containing polysaccharides, oligosaccharides, glycoproteins, and synthetic polymers on their surface exhibit even longer circulation time. To summarize, the liposomes highlighted above show enhanced blood circulation time, improved biodistribution, and greater stability and efficiency 19, 27. However, the benefits of nanovectors are not limited to spherical carriers. The merits of more complex structures will be presented below.

### Dendrimers

Dendrimers (dendrons) comprise three main parts: a repeated unit connected to the central core, the core, and functional end groups 5, 28. Different dendrimer-based nanocarriers have been utilized for cancer therapy. Currently, poly(amidoamine) (PAMAM), peptide dendrimer, polylysine, poly-l-lactide, poly-caprolactone, poly(ethylene glycol), and poly(propylene imine) are most commonly employed 29. In particular, anticancer agent-loaded dendrimers combined with iron oxide or gold nanoparticles (AuNPs) have received great attention in cancer treatment 30. Attaching targeting molecules to dendrimers can enhance the cancer-specific delivery of antitumour agents and decrease the unwanted effects. Antigens and ligands such as folate, dextran, and galactose are typically used for this purpose and are associated with the cationic cytotoxicity of dendrimers 31, 32. Light, temperature, pH, and other stimuli have been used to develop stimulus-responsive dendrimers 33.

**Figure 3:**
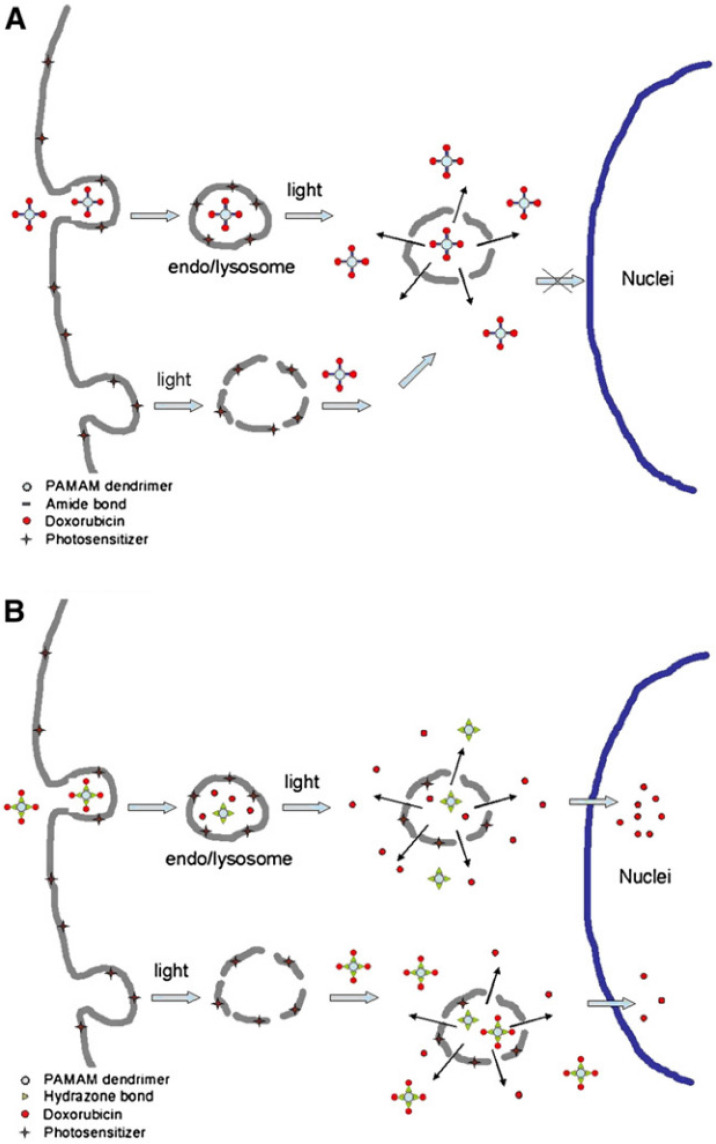
A suggested model for the cellular uptake of (A) poly(amidoamine) (PAMAM)-amide-doxorubicin and (B) PAMAM-hydrazone-doxorubicin in light-exposed Ca9-22 cells. Nuclear accumulation of doxorubicin is denoted by arrows 34. Reproduced with permission. Copyright Elsevier (2007).

PAMAM dendrimers conjugated to doxorubicin have been used as efficient pH-responsive nanocarriers to treat oral cancer *in vitro* 34. pH-insensitive PAMAM-amide-doxorubicin conjugates remain intact after liberation from vesicles and do not enter the cell nucleus (Figure 3A) 34. As shown in Figure 3B, doxorubicin is released due to the cleavage of hydrazone bonds in endosomes/lysosomes. Upon light exposure, the endosomal/lysosomal membrane is broken up, and doxorubicin translocates to the nucleus. However, earlier light exposure leads to premature breakdown of the endosomal/lysosomal membrane. Consequently, there is less cleavage of hydrazone bonds, diminishing doxorubicin release and translocation to the nucleus.

**Figure 4:**
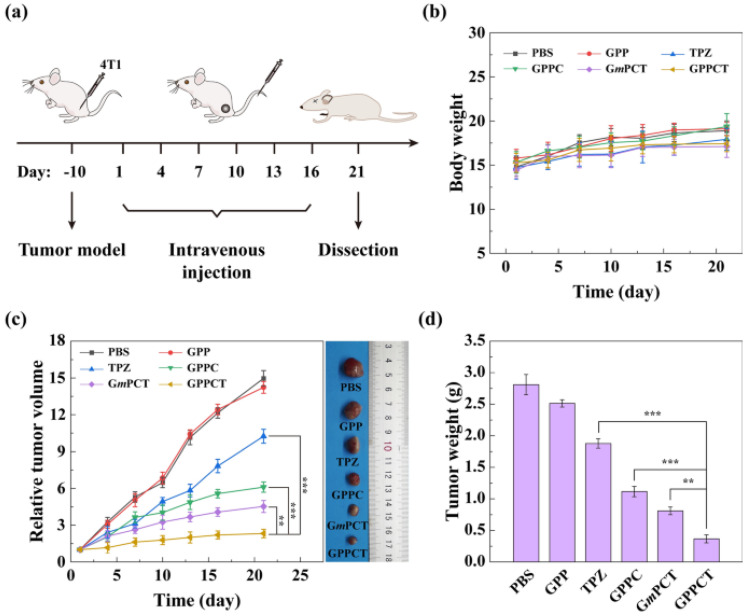
*In vivo* treatment of tumour-carrying mice treated with phosphate-buffered saline (PBS), G5.NHAc-PEG-PBA (GPP), G5.NHAc-PEG-PBA@Cu(II) (GPPC), G5.NHAc-PEG-PBA@Cu(II)/TPZ(CPPCT), G5.NHAc-mPEG@Cu(II)/TPZ (GmPCT), or tirapazamine (TPZ). (a) Treatment strategy. (b) Body weight. (c) Relative tumour volume. (d) Tumour weight 35. Reproduced with permission. Copyright Springer (2022).

Another study evaluated the therapeutic effect of PEG-phenylboronic acid (PBA)-modified generation 5 (G5) PAMAM dendrimers *in vivo* in 4T1-tumour transplanted BALB/c nude mice (receiving phosphate-buffered saline [PBS], G5.NHAc-PEG-PBA [GPP], G5.NHAc-PEG-PBA@Cu(II) [GPPC], G5.NHAc-PEG-PBA@Cu(II)/TPZ [CPPCT], G5.NHAc-mPEG@Cu(II)/TPZ [GmPCT], or tirapazamine [TPZ]) 35. As depicted in Figure 4C, pure nanocarriers (GPP) could not inhibit tumour growth. Despite the tumour inhibitory effects of TPZ, its low uptake rate and fast metabolism precluded its potential therapeutic effects. On the contrary, the GPPC group acted more efficiently in a target-specific manner due to its uptake and accumulation in the tumour niche. Figure 4D shows the tumour weights for each group.

### Micelles

**Figure 5:**
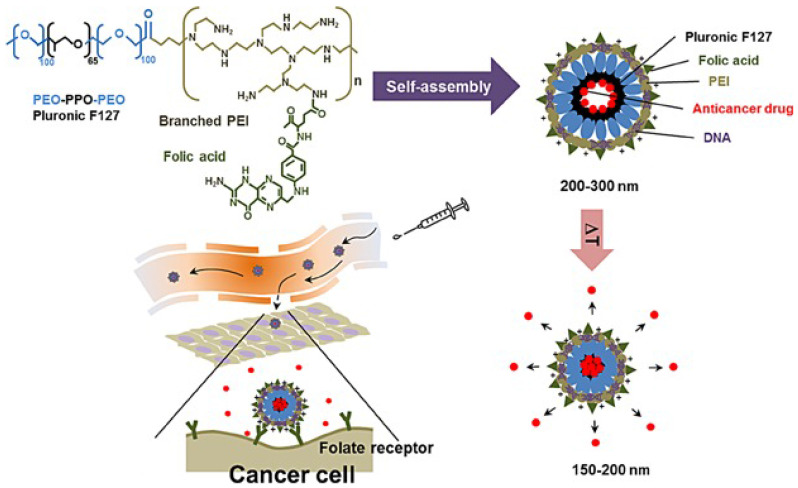
The temperature-responsive micelle-based delivery system for co-delivery genes and anticancer drugs 39. Reproduced with permission. Copyright Wiley (2015).

Another way to shape nanovectors is to use micelles, which can assume a broad spectrum of shapes. Polymeric micelles have recently gained significant attention and have become one of the most well-examined nanocarriers in cancer theranostics. Micelles (10-100 nm) are self-assembled structures in water with a hydrophobic core and a hydrophilic corona that can accommodate hydrophobic medications in their core 36, 37. Different ligands, including antibodies, aptamers, folate, and carbohydrates, have been implemented to decorate the micellar surface. Ultrasound, enzymatic, temperature, and pH alterations have been used to provide stimulus-responsive micellar drug delivery systems 38. The intracellular uptake of micelles can be enhanced by attaching several functional groups to the hydrophilic end of the micelles. For instance, Seo et al. 39 constructed a temperature-responsive micelle-based system for the co-delivery of genes and anticancer drugs (Figure 5), which contains crosslinked polyethyleneimine-modified micelles modified with a targeting molecule (FA). This multifunctional nanosystem exhibited no toxicity. Genes and short interfering RNAs (siRNAs) have also been efficiently delivered by polyion complex micelles 40. Amphiphilic copolymer-based micelles have been used for concomitant cisplatin and paclitaxel delivery and have notably increased loading efficiencies 41. Moreover, a papain-functionalized, mucopermeating, thiolated redox micelle has been applied for the targeted delivery of paclitaxel. This micelle-based nanoformulation inhibited the P-glycoprotein efflux pump and enhanced the bioavailability and penetration of the drug 42.

It has to be stressed that the utility of nanovectors for cancer treatment is not limited to biological molecules arranged on the nanoscale. Over the past decades, a plethora of nanomaterials has been discovered that can contribute toward solving this issue.

## Nanomaterials as nanovectors

### Carbon Nanotubes (CNTs)

CNTs are carbon-based cylindrical structures with an open core that might serve as valuable nanocarriers in cancer treatment 43. Single-walled carbon nanotubes (SWCNTs) and multi-walled carbon nanotubes (MWCNTs) are classified based on the number of graphene layers utilized to construct them. Unique physicochemical properties of CNTs, such as intracellular bioavailability, an ultra-high aspect ratio, and high cargo loading 44, 45, make them applicable in this area. They are especially useful as CNTs have a longer residence time in lymph nodes than liposomes 43. Thus, they are promising nanocarriers for treating lymph node tumours 46. In this context, Yang et al. 46 developed folic acid-functionalized MWCNTs containing superparamagnetic Fe_3_O_4_ nanoparticles conjugated to cisplatin. The authors used an external magnet to guide the MWCNTs to the lymph node 46. They found that the drug was released in the tumour cells for several days 46, making the treatment approach successful.

**Figure 6:**
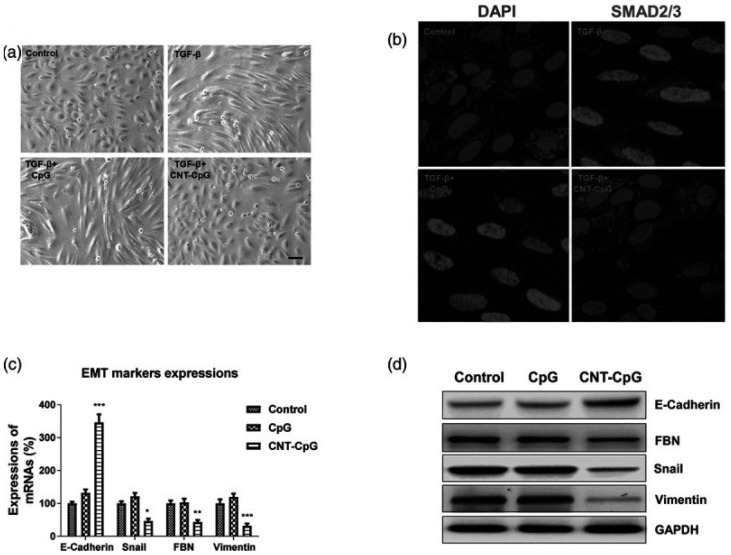
Carbon nanotube conjugated to CpG (CNT-CpG) inhibits the transforming growth factor-beta (TGF-β)-induced epithelial-to-mesenchymal transition (EMT) of colon cancer cells. (a) Morphological alterations in HCT116 cells. (b) Treated cells were stained with DAPI and an anti-SMAD2/3 antibody. (c and d) Messenger RNA (mRNA) and protein expression of EMT marker genes were detected by real-time polymerase chain reaction and western blot, respectively 52. Reproduced with permission. Copyright Wolters Kluwer Health (2020).

Furthermore, functionalized CNTs have been utilized as nanovectors in gene therapy to deliver biomolecules such as aptamers, microRNA (miRNA), siRNA, and DNA 45, 47. They have demonstrated high therapeutic efficacy 48, 49. Besides that, CNTs have been used in cancer immunotherapy 50. Recent experimental studies have revealed that gliomas can uptake CNT-conjugated CpG complex (CNT-CpG), which showed tumour suppressor effects *in vitro* and *in vivo* 51. Treatment of colon cancer with CNT-CpG significantly inhibited the epithelial-to-mesenchymal transition (EMT) signaling pathway and translocation of transcription factor SMAD2/3 51. Additionally, there was a substantial decrease in the expression of EMT-related markers such as vimentin, Snail, and fibronectin 51. In another study, epithelial cell marker E-cadherin expression increased *in vivo* in xenograft mice tumours, while vimentin was downregulated following CNT-CpG treatment (Figure 6) 52.

### Inorganic nanoparticles

Similarly to CNTs, nanoparticles with different elemental compositions, structures, and properties show encouraging potential for several biomedical applications comprising performances as nanocarriers for drug/RNA/DNA/gene delivery, as contrast agents for bio-imaging techniques, or radio-enhancing/photothermal therapeutic agents. Suitably coated gold, Al_2_O_3_, ZrO_2_, SiO_2_, MnO, Fe_3_O_4,_ and Fe_2_O_3_ nanoparticles with sizes between 10 and 50 nm are not only biocompatible, non-toxic and chemically stable but also provide sufficiently large surfaces for attaching different and complementary and mutually enhancing functionalities, making them often more stable than organic materials 53.

Gold nanoparticles (AuNPs) exhibit unique features such as localized surface plasmons, adequate biocompatibility, and high affinity for thiols when used as vectors 54. Multiple synthesis strategies are used to adjust their morphological properties and surface functionalization. Turkevitch and Brust-Schiffrin 54 synthesis techniques are used to prepare gold nanospheres. Whereas Turkevich's method utilizes gold acid as a precursor and sodium citrate as a reducing agent and surfactant, Brust-Schiffrin's synthesis of thiolated AuNPs occurs via a two-phase procedure with sodium borohydride for the reduction of gold acid and tetraoctylammonium bromide as phase transfer agent. The direct use of as-introduced functional thiols and pre-coordinated thiolate ligands facilitates these nanoparticles to act as nanovector 55. Chemically modified AuNPs exhibited specific cytotoxicity to cancerous cells while sparing healthy cells. In this context, the dose, the nature of the ligand, the sizes of the AuNPs, and the administration route determine the cytotoxicity, which should be taken into account while designing AuNP-based nanovectors for cancer treatment 54.

Due to their competitive performance, AuNPs have attracted increasing attention in developing different nanocarriers for drug delivery, tumour sensing, and photothermal cancer therapy 56. They exhibit several unique features that make them promising theranostic cancer agents. The inactivity of AuNPs towards biological systems is among their most prominent advantages compared with metal-oxide-based nanoparticles 57. Besides that, AuNPs perform excellently as plasmonic heating agents by converting near-infrared (NIR) light into heat and are thus best suited for application in photothermal therapy of malignancies. AuNPs with proper sizes and shapes exhibit no phototoxicity, so they may be considered biocompatible agents 58, 59.

Concomitantly, the implementation of high atomic number (Z) materials to increase sensitivity to X-ray irradiation has been studied for over half a century. High-Z materials do emit not only secondary X-rays but also Auger, Compton, and photo-electrons which induce DNA damage and ionize water, producing reactive oxygen species (ROS). These phenomena may be used for cancer treatment 60. *In vitro* and *in vivo* studies confirmed that AuNP-based radiation therapy eradicates tumuor cells with high efficiency 61, 62.

**Figure 7:**
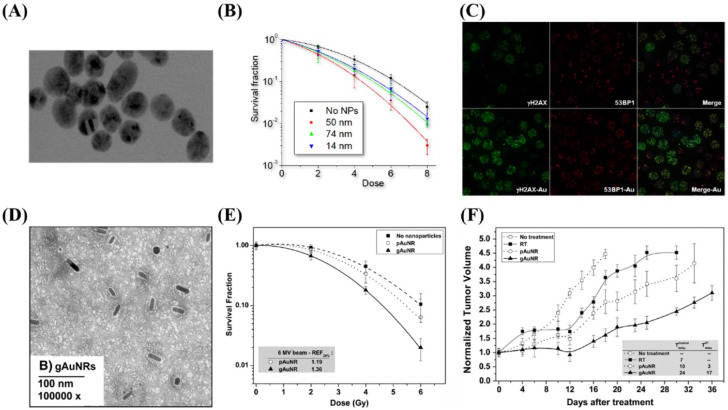
The use of gold nanoparticles (AuNPs) to enhance the lethal impact of X-rays on tumour tissue. (A) A micrograph of spherical AuNPs. (B) The clonogenic assay showed that the largest effect occurred with 50-nm AuNPs. (C) Confocal micrograph showing the uptake of AuNPs. (D-F) Increased radiosensitization of Graphene nanoribbons (GNRs) *in vitro* in prostate cancer cell lines and *in vivo* by measuring the tumour volume. Reproduced with permission from 67. Copyright Elsevier, 2015.

Enhancing the X-ray irradiation dose by AuNPs was first established in an *in vivo* experiment using 1.9 nm AuNPs 63. In this study, 30 Gy X-ray irradiation significantly reduced the volume of mice's subcutaneous tumours. However, Hainfeld et al. utilized high concentrations of AuNPs (2.7 g/kg), a dose that is not clinically feasible. AuNPs with sizes smaller than 2 nm were observed to invade deep-lying tumours, which boosted the local X-radiation dose to maximum therapeutical efficacy. In the case of successful internalization of AuNPs, radiosensitization is feasible even at low concentrations 64, 65. Chithrani et al. 66 reported increased radiosensitization at 6 MV by means of 50-nm spherical AuNPs (Figure 7).

Furthermore, AuNP-conjugated methotrexate exhibits higher cytotoxicity towards tumour cell lines compared with free methotrexate. Doxorubicin bound to AuNPs by acid-labile bonds shows increased toxicity to breast cancer cells line 68-70. It was also reported that the conjugation of AuNPs with fluorescent heparin as a targeting agent creates a promising material cancer diagnosis 71, 72. One should also keep in mind that AuNPs provide enhanced superoxide radical anion generation and therefore induce cell death directly 72.

To make this approach more sustainable, herbal or biogenic strategies have also recently been applied to produce AuNPs. For example, AuNPs were prepared from *Abies spectabilis* and as-produced material exerted an anticancer effect on bladder cancer 73. AuNPs are cytotoxic for bladder cancer cell lines (T24) by stimulating apoptosis, nuclear DNA fragmentation, and DNA injury 74. Upon NIR or visible light irradiation, AuNP exhibit localized surface plasmon resonance (LSPR) that is highly sensitive to environmental changes, a phenomenon that enables applications in imaging techniques for cancer diagnosis.

In addition, suitably functionalized AuNPs allow the detection of abnormal sequences of amino acids via hybridising techniques. Antibody-conjugated AuNPs facilitate tumour-targeting and thereupon accumulation of the AuNPs in cancerous tissue. AuNPs when acting as drug nanocarriers can perform either in active or passive way 75. In active vectorization, AuNPs bind to overexpressed receptors on the plasma membrane of cancer cells and are subsequently endocytosed. In passive vectorization, drugs are conjugated to AuNPs. In summary, AuNPs are among the most prevalent type of nanoparticles to modulate cancer because they act as multifunctional and safe materials in cancer theranostics 76.

Silver nanoparticles (AgNPs) have also shown great potential due to their antimicrobial, antiviral, and anticancer nature. Due to these properties, AgNPs are very widely used. It has been reported that AgNPs induce cytotoxicity via apoptosis and necrosis in different cancerogenic cells and exhibit results against secondary effects of current therapies (e.g. deoxyribonucleic acid (DNA) damage, generation of reactive oxygen species, increasing leakage of lactate dehydrogenase). In addition, AgNPs have shown some unique properties, such as ten times greater light scattering cross-section than AuNPs 77, making these particles very interesting for their biosensors 78, 79. AgNPs can also show great potential as photo-controlled drug delivery vectors due to their more significant extinction coefficient and blue-shifted plasmon resonant peak 80.

Besides nanostructures from noble elements, simple metal oxide NPs (MONPs) also exhibit favorable characteristics since they are chemically stable and easy to process to the desired size, shape, and porosity. In addition, they may be incorporated into hydrophobic and hydrophilic systems and can be facilely functionalized with various molecules via covalent or electrostatic binding 81. MONPs can be classified based on the dimensionality into 0- (e.g. nanocluster, quantum dots), 1- (e.g. nanorods, nanowires), 2- (e.g. nanoshets), and 3-dimensional (e.g. scaffolds) materials 82, which further increases their application potential as changing the shape dramatically modifies the biological properties. What is important, many MONPs, for example, TiO_2_, ZnO, ferric oxide (Fe_2_O_3_), and ferrous oxide (Fe_3_O_4_), appear to be safe for mammals 82. They perform well as nanocarriers with excellent delivery efficacy, as they can invade the body via respiration, ingestion, skin infusion, or even direct injection 81.

### Mesoporous Silica Nanoparticles (MSNs)

Another vital factor affecting biological performance is the material's porosity. Mesoporous silica nanoparticles (MSNs) possess an exceptional potential as versatile nanoplatforms for cancer theranostics 83-86 because of high biocompatibility, uniform pore size distribution, large pore volume, substantial surface area, and the capability for further chemical modifications. The mesoporous surface of the silica nanoparticles provides exceptional opportunities for drug loading and its stable release 87, 88. Mobil Composition of Matter 41 (MCM-41) is the most studied MSN for cancer treatment, which simultaneously is the most promising silica-based material for this application 89. It was found that the performance of MSNs can be extended by coating them with PEG, thus making long-circulating nanocarriers 90.

**Figure 8:**
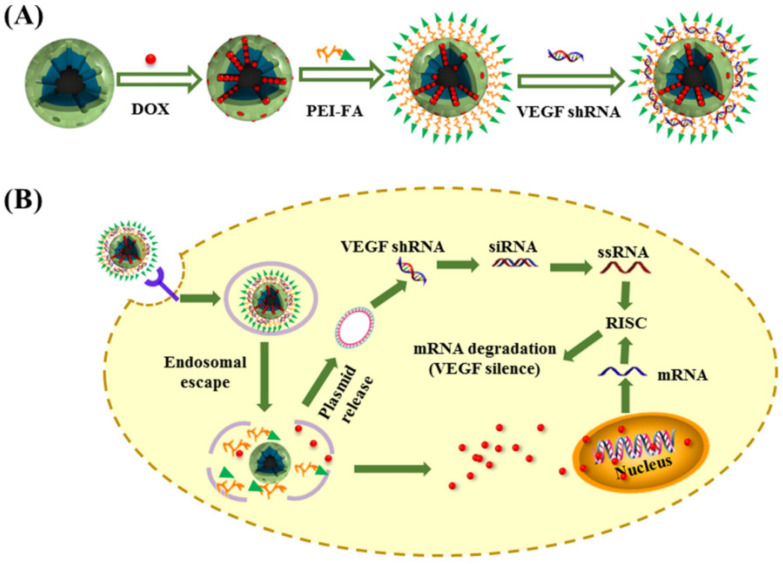
Construction of a multi-component cancer treatment agent based on MSNs depicted in the top left corner. (B) The mechanism of action *in vivo*. Reproduced with permission from 91. Copyright American Chemical Society (2016).

Folate-modified MSNs have shown significant cytotoxic effects against human breast and cervical cancer cells 91, 92. Li and colleagues reported the construction of MSN-based nanocomplexes composed of MSNs, doxorubicin (DOX), polyethyleneimine (PEI)-conjugated folic acid, and Vascular Endothelial Growth Factor shRNA that enabled co-delivery of chemotherapeutics and nucleic acid drugs to improve cancer treatment (Figure 8). MSNs are also suitable carriers for nucleic acid-guided treatments and nucleic acid delivery to tumour cells 93-97. They have recently been applied in photodynamic and photothermal therapy of different cancer types, reaching appreciable performance 97.

### Superparamagnetic Iron Oxide Nanoparticles (SPIONs)

**Figure 9:**
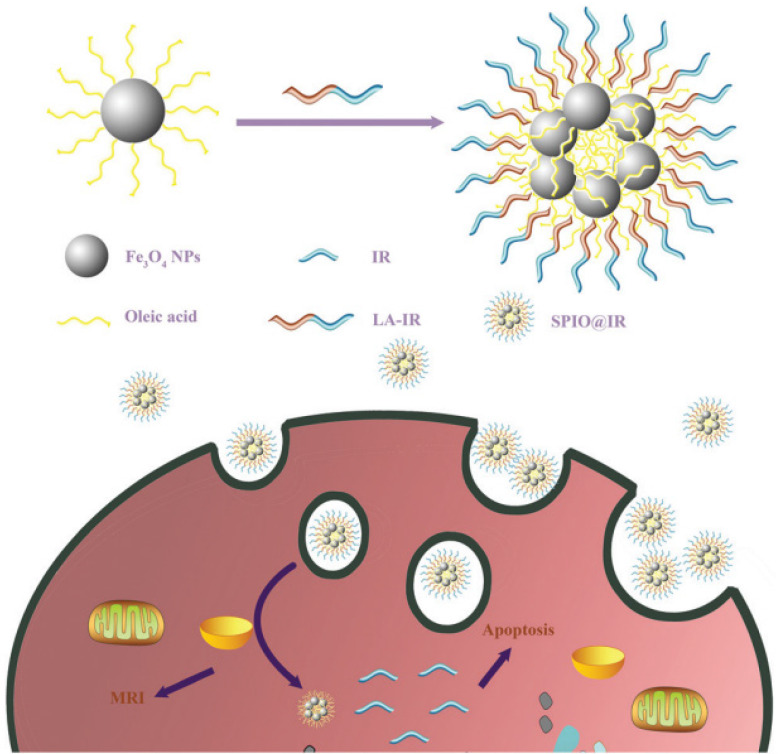
Generation of bio-compatible SPIONs and their interaction with cells. Abbreviations: Fe_3_O_4_ NPs, superparamagnetic iron oxide nanoparticles; IR, irinotecan; LA-IR, amphiphilic lauric acid-irinotecan prodrug; SPIO@IR, LA-IR inserted SPIO prodrug. Reproduced with permission from 98. Copyright Royal Society of Chemistry (2018).

An important aspect of any treatment is the ability to direct it to the right location. One of the types of materials that can be guided and accumulated in tumour tissue by applying an external magnetic field are superparamagnetic iron oxide nanoparticles (SPIONs), which have recently received considerable attention from the scientific community 98. Suitably functionalized SPIONs are biocompatible, and they have high application potential due to their properties. For example, they are excellent contrast agents for magnetic resonance imaging (MRI) and can acs as magnetic hyperthermia agents under an altering magnetic field 99. The scheme below displays how SPIONs can be used for nanotheranostics in cells (Figure 9).

Because bare SPIONs agglomerate in aqueous solutions, they must be stabilized to form stable aqueous colloids 100. Solar and co-workers 101 reported coating SPIONs with poly(3-hydroxybutyrate-co-3-hydroxyvalerate) (PHBV) as a biodegradable and biocompatible polymer to avoid toxicity while preserving all of its potential applications. Recent studies in the realm of drug delivery and tissue engineering have confirmed that PHBV 101 is a cost-effective material that shares similar physicochemical properties with the majority of widely used polymers 102-104.

The size of a SPION-based drug delivery system should be 10-100 nm for *in vivo* application to avoid renal clearance and RES 105. To extend their applicability, SPIONs can be conjugated with different kinds of biocompatible materials such as liposomes, polymers, and viral vectors. Non-viral drug delivery via surface-modified SPIONs has recently undertaken outstanding developments 106, 107, and they can specifically reach tumour sites 108. Moreover, the use of targeting moieties conjugated to SPIONs has resulted in a significant reduction in the required and off-target effects 109.

### Quantum Dots (QDs)

**Figure 10:**
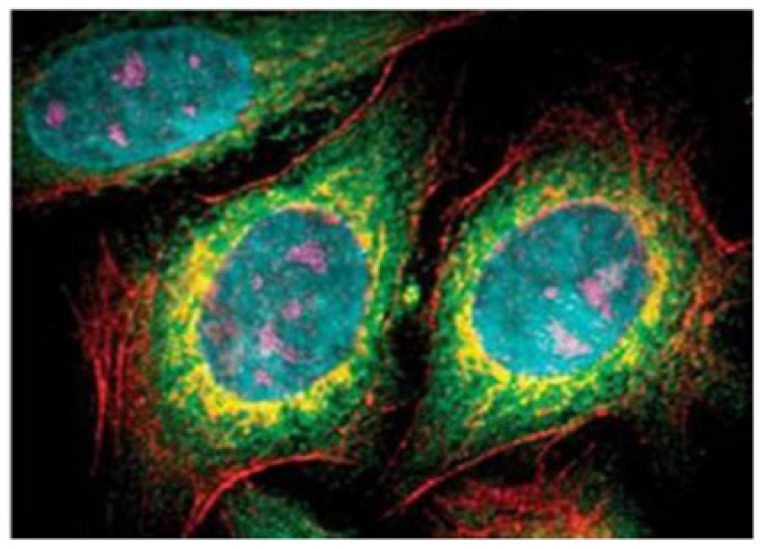
The multi-color imaging of fixed human epithelial cells using five types of QDs. Adapted with permission 110. Copyright Springer (2015).

There are other shapes of nanoparticles that can also be helpful in cancer theranostics. QDs, including Si QDs, ZnO QDs, and ZnS QDs, possess interesting optical, electrical, and fluorescence properties 110. Fig. 10 shows how multi-color imaging of fixed human epithelial cells can be accomplished using five different color QDs.

With appropriately modified surfaces, water-soluble ultra-small QDs (2-4 nm) can be produced 111 and employed in nanocarriers to visualize tumour tissue and concomitantly release a drug in the desired region. Commercially available QDs comprise three components: a core (a semiconductor material), a shell, and functional ligands. The core and surrounding shell are semiconductor materials, and caps enclose double-layered QDs 112. Functionalising QDs with biocompatible polymeric materials or targeting moieties can substantially improve their performance 113, 114. Furthermore, QDs escape from the RES and renal clearance due to their small size, so their blood circulation time increases 80.

Senel and colleagues 115 developed N-doped graphene quantum dots as cost-effective materials that show antioxidant and anti-inflammatory effects. According to their results, N-doped graphene QDs could be linked to DNA via electrostatic interactions or intercalation. The graphene QDs easily penetrate cancer cells and eradicate them with high efficiency. siRNA can be integrated into the QD structure to produce agents that are effective in cancer treatment even at low doses 115. Akbarzade et al. 116 synthesized an aptamer-functionalized mesoporous silica-coated QD. The nanohybrids are suited for MRI and fluorescent imaging. In another study, researchers utilized GQDs for drug delivery in cancer treatment 117. Javanbakhta et al. 118 obtained nano-carboxymethyl cellulose (CMC)/GQD hydrogels with superb mechanical and rheological properties. CMC/GQDs showed high permeability, negligible toxicity, and exerted a prolonged doxorubicin release profile. Antibody-linked graphene QDs nanoparticles have been used to deliver cisplatin and targeted cellular imaging 119. In addition, Fe_3_O_4_@SiO_2_@GQD-FA nanoparticles have synergistic effects with doxorubicin in Hela cells 120. Overall, the large volume of research on QDs for cancer theranostics validates their substantial application potential for this purpose.

### Nanoshells, Nanowires, and Cantilevers

**Figure 11:**
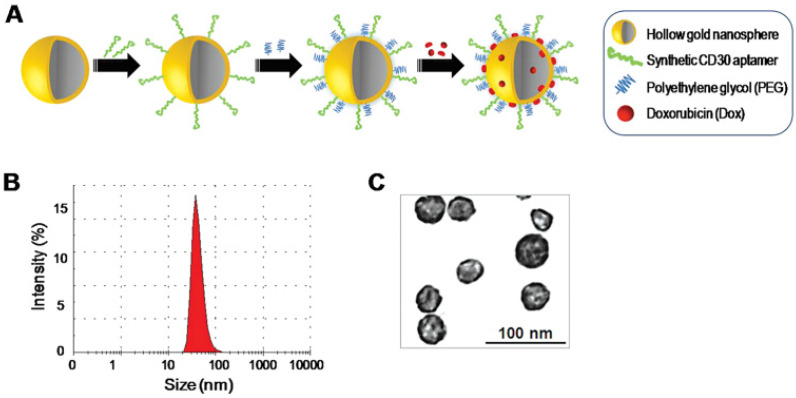
(A) formation of gold nanosphere-based agent, (B) determination of size by dynamic light scattering, (C) TEM micrographs of the obtained material. Adapted with permission 124. Copyright Wiley (2013).

Other more complex shapes of nanoparticles also have their utility for cancer theranostics 121. For instance, nanoshells exhibit increased permeation and retention effects due to their small size (Figure 11), so they accumulate preferentially at the tumour site 121. Results from *in vivo* studies have demonstrated that nanoshell administration could be considered a low-toxic approach 122. Conjugation of nanoshells with biomolecules facilitates the recognition of the tumour location and spares healthy cells. The ability of nanoshells to absorb different energy sources, such as light and radio frequency, has turned them into potent tumour cell killers 123.

Furthermore, some nanoshells absorb NIR light, and hence they can act as a plasmonic heater to produce the heat required for photothermal therapy of specific tumour cells. Liang et al. 123 synthesized doxorubicin-functionalized gold nanoshells that release the drug into the tumour tissue in a pH-dependent manner. Gold nanoshells can also be conjugated to different targeting moieties that enhance cellular uptake of the drug. In this context, aptamer-functionalized doxorubicin-loaded gold nanoshells have been shown to bind specifically to CD30-overexpressed lymphoma cells. The acidic pH of lysosomes triggers drug release from this system 124.

Simultaneously, microfluidic channels can be used to house nanowires for sensing molecular signatures of analytes that flow through the channel and relay this information to the external electrodes. Nanowires are highly specific and selective sensing devices that can detect the presence of altered genes in a distinct cancer cell type 125. Peng et al. 126 reported silicon nanowires developed to deliver docetaxel as an ultra-performing nanovector. This nanocarrier had an ultra-high drug-loading capacity and exhibited a substantial anticancer effect both *in vitro* and *in vivo*. Kuei-Chang et al. 127 developed the first PEGylated copper nanowire for photothermal treatment. It converts NIR light to heat with good flexibility and reproducibility. In addition to the effect of this nanowire *in vitro*, its intra-tumoural injection significantly impaired colon cancer growth.

Lastly, cantilevers are lithographic structures that generate flexible microscopic beams and can be coated with different molecules to provide a platform for detecting distinct disease markers, including cancer 128. In other words, the tip mounted at one end of a cantilever can be attached to diagnostic molecules that, in turn, may detect specific DNA-binding proteins expressed by certain cancer types. Wu et al. 128 developed a nanocantilever that can detect PSA at levels lower than the clinically relevant threshold value. This method is cost-effective, highly sensitive, and potentially more effective than enzyme-linked immunosorbent assay (ELISA).

## Extracellular vesicles (EVs)

**Figure 12:**
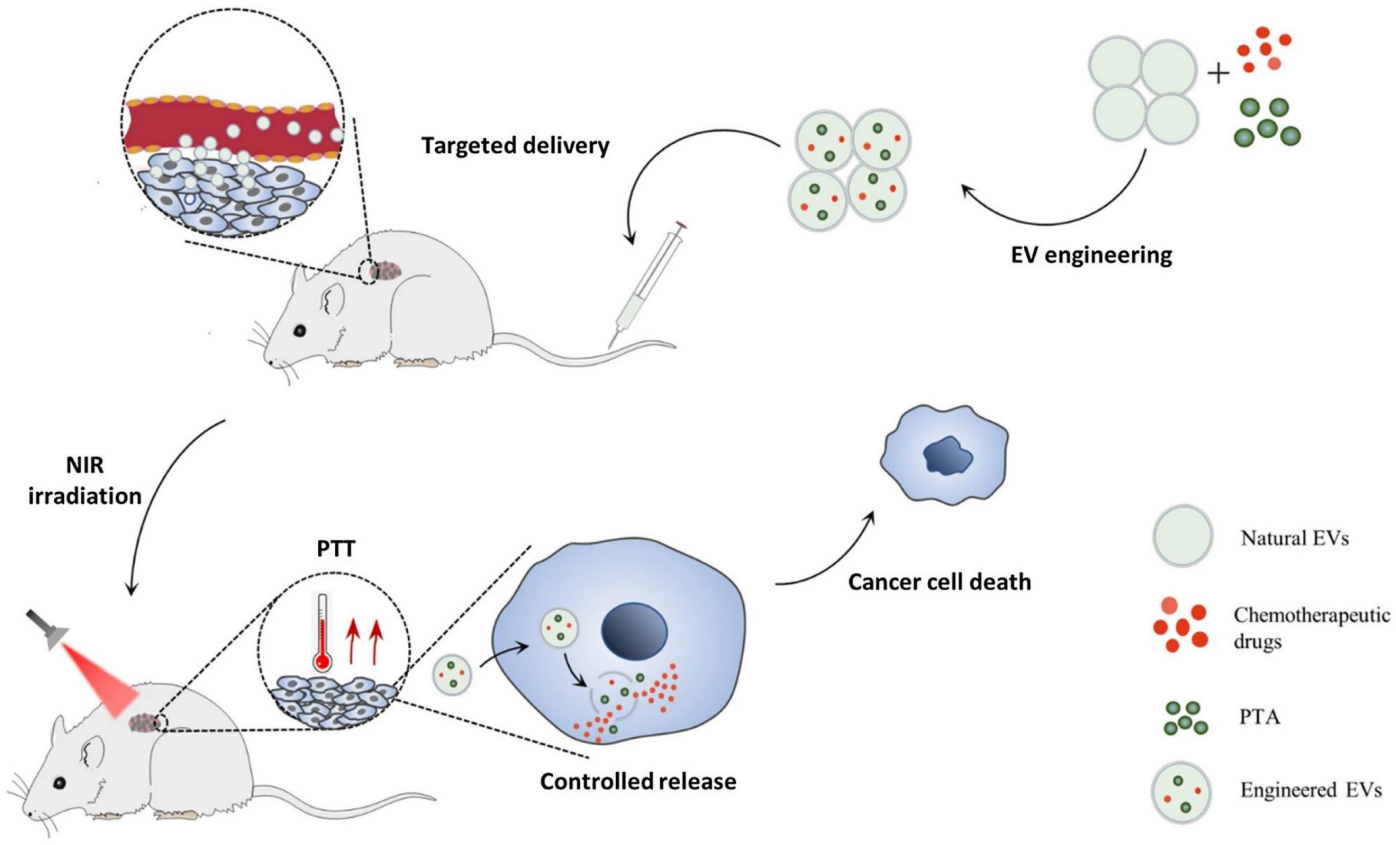
Chemo/photothermal therapy by engineered extracellular vesicles (EVs) that co-deliver drugs and photothermal agents (PTAs) to tumour sites 129. Reproduced with permission. Copyright Springer (2022).

EVs are a heterogeneous group of lipid bilayer membrane-enclosed vesicles that provide cell-cell communication via the transmission of different cargo. Small extracellular vesicles (sEVs) have gained importance as innovative, safe, natural, and efficient delivery systems in the treatment of diverse diseases, in particular cancer. Figure 12 depicts a recently engineered EV vehicle for the co-delivery of photothermal agents (PTAs) and drugs. Different types of sEVs are discussed in the following subsections.

### Cancer Cell-Derived sEVs

Cancer cell-derived sEVs have been considered appealing delivery vehicles of different anticancer agents because they accumulate in tumours due to the cellular tropism of sEVs 130, 131. Thus, autologous sEVs are mainly used and will be discussed here. Cancer cell-derived sEVs can transfer tumour antigens to dendritic cells, stimulating the immune response against cancerous cells 132. Using special sEVs as drug carriers substantially enhance the efficacy of the pharmaceutical in different cancer types 133, 134. For example, the incubation of prostate cancer cell-derived EVs with paclitaxel significantly elevated the cytotoxic effect of paclitaxel in autologous parental prostate cancer cells after endocytosis 133. In another study, gemcitabine-loaded pancreatic cancer cell-derived sEVs exhibited a more significant accumulation of drugs after intravenous injection to pancreatic cancer cell xenograft mice 134. Emam et al. 135 investigated the cellular uptake of sEVs derived from colorectal C26- and melanoma B16BL6 cells by each cell type. They showed that the uptake of sEVs is larger in donor cells than in recipient cells. Kim et al. 136 confirmed the cellular tropism of sEVs. They evaluated the cellular uptake of ovarian cancer cell line (SKOV3)-derived sEVs by SKOV3 and epithelial cell (HEK293). These sEVs accumulated more in the SKOV3 cell line to a larger extent 136. Although using autologous sEVs has been considered a safe technique, one should be aware of the immune-inhibitory effects of cancer cell-derived sEVs 134. The origin of sEVs and their cargo determine their inhibitory or stimulatory effects on the immune system. Moreover, cancer cell-derived sEVs might also increase tumour cell growth and metastasis and induce drug resistance 133. Therefore, additional studies are required to evaluate the possible tumour stimulatory effects.

### Mesenchymal stem cell (MSC)-derived sEVs

MSCs are multipotent stem cells with extensive use in regenerative medicine due to their exceptional properties, such as self-renewal and capacity to differentiate into targeted lineages 137, 138. MSC-derived sEVs have attracted great attention for their therapeutic potential in cancer treatment, in addition to their use in regenerative medicine 138. These sEVs can also carry pharmaceutical agents. The expression of distinct receptors for humoral factors, including cytokines, growth factors, and chemokines in the tumour niche, enables them to develop tumor-suppressing effects 139. Moreover, the presence of inflammatory factors in the tumour microenvironment can stimulate the migration of MSCs into cancerous regions 140. MSC-derived sEVs exhibit paracrine effects by transmitting their properties to recipient cells. Therefore, they can serve as efficient nano-sized vectors for treating cancer 141. Bone marrow, placenta, adipose tissue, and amniotic fluid can be the source of sEVs 142. Previous reports have shown the therapeutic capacity of sEVs in treating therapy-refractory graft-versus-host disease 143. Moreover, their effectiveness in treating acute ischaemic stroke is being evaluated in an ongoing clinical trial (NCT03384433). MSC-derived sEVs containing paclitaxel have also been shown to suppress tumour growth 144.

**Figure 13:**
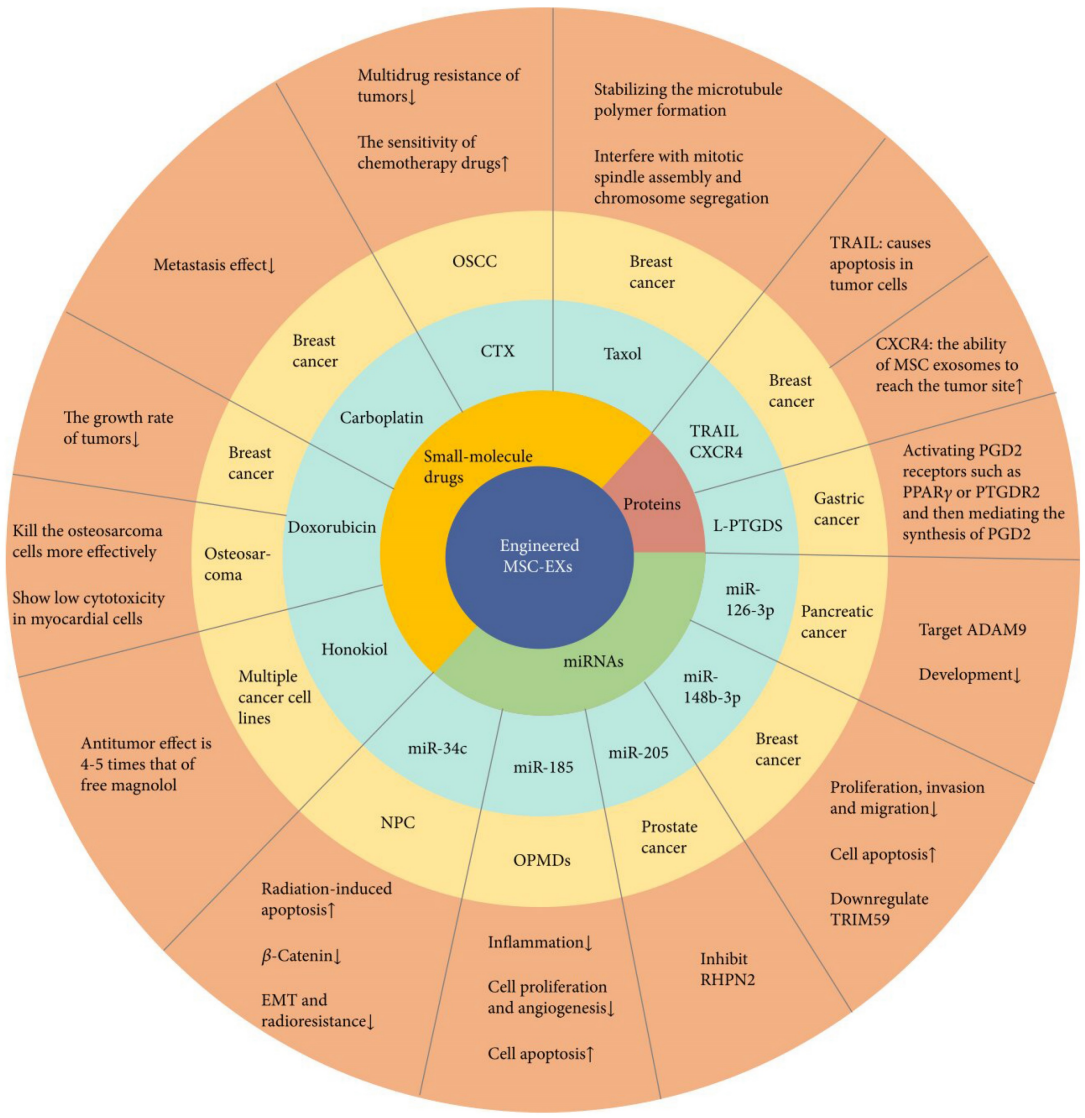
Engineered mesenchymal stem cell exosomes (MSC-EXs) for cancer treatment. These structures carry microRNAs (miRNAs), small-molecule drugs, and proteins 151. Reproduced with permission. Copyright Hindawi (2021).

Short RNA sequences, small-molecule drugs, and proteins are the main types of cargo delivered by engineered exosomes 145. However, few studies have addressed the successful loading of proteins, which might be due to their high molecular weight, the uncertain mechanism of protein sorting by exosomes, and the absence of effective loading techniques 146, 147. The first report on protein loading into EVs for cancer treatment was by Mizrak et al. 148 in 2013. They prepared microvesicles carrying a suicide mRNA/protein that hampers the growth of a schwannoma tumour. Later, they developed a method for protein loading based on the evolutionarily conserved late-domain (L-domain) cascade. Using this technique, they loaded WW tag-labeled Cre recombinase into exosomes 146. In another study, researchers transfected MSCs with tumour necrosis factor-related apoptosis-inducing ligand (TRAIL) and chemokine receptor type 4 (CXCR4) via exosomes. They showed synergistic effects with carboplatin *in vivo* 149. Exosomes have been utilized to deliver lipocalin-type prostaglandin D synthase (L-PTGDS) to gastric cancer cells 150. L-PTGDS overexpression has been associated with reduced gastric cancer growth, reduced formation of tumour clones, and reduced migration of cancer cells, and thus can act as a possible therapeutic agent. L-PTGDS shows its potential therapeutic effects by mediating the production of prostaglandin D2 and stimulating PGD2 receptors, including Peroxisome proliferator-activated receptor-gamma on the surface of tumour cells. Figure 13 illustrates the role of exosomes for the delivery of RNA, drugs, and protein according to previous research activities 151.

Although a few clinical studies have been devoted to anticancer effects of MSC-derived sEVs, these effects are still debated because MSCs *per se* can increase tumour cell proliferation and induce angiogenesis 138. For example, MSCs can boost breast cancer metastasis 152. Therefore, the use of sEVs harvested from MSCs should be carefully considered. So far, researchers have noted that the cell cultivation platform, tumour model, and tumour microenvironment might determine the protumour or antitumour effects of MSC-derived sEVs 153.

### Immune Cell-Derived sEVs

Immune cells such as macrophages, T cells, dendritic cells, and natural killer (NK) cells also provide sEVs that could be used for drug delivery 154. These cells can naturally regulate several antitumour immune responses, enhancing therapeutic efficiency. Dendritic cell-derived sEVS are promising candidates for nanovectors for cancer therapy, as the approval of several clinical trials has confirmed their efficacy, in particular, for antitumour vaccination 155, 156. Nevertheless, it should be noted that these sEVs might lead to immune inhibition and thus cancer metastasis 157.

**Figure 14:**
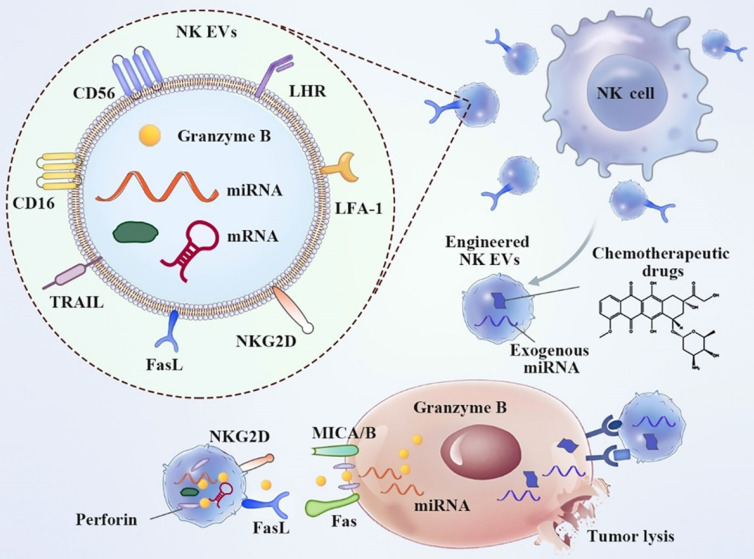
Typical properties and anticancer implementation of natural killer (NK) cell-derived extracellular vesicles (EVs). NK EVs bind cancer cells via NKG2D-MICA/B and promote cytotoxicity by releasing their cytotoxic protein cargo. In addition, engineered NK EV-coated nanoparticles have been used to deliver anticancer agents 164. Reproduced with permission. Copyright Frontiers (2021).

NK cells are innate immune effectors that play a pivotal role in organ immunosurveillance, microorganism-related infections, and cancer 158. Human leukocyte antigen (HLA)-linked inhibitory receptors expressed on membranes stringently control the activity of NK cells under steady-state conditions 159. NK-derived EVs encompass NK cell surface receptors (Figure 14). EVs released from active NK cells can induce apoptosis in cancer cells. On the contrary, NK ligand-bearing cancer cells decrease the expression of active receptors, including NKG2D, and block NK cell degranulation, leading to compromised toxicity and diminished levels of lytic proteins 160. Many cytotoxic proteins exist, including granzyme, perforin, FasL/CD178, small antimicrobial peptides, granulysin, and TRAIL 161. Direct killing pathways are then activated and target different types of cancer 162, 163.

As mentioned above, the majority of surface receptors are expressed on immune cell-derived EVs and their parental cells, which allow them to detect target cells and liberate their cargo. For example, proteins involved in the co-stimulation of T cells (CD80, CD86, and Intercellular Adhesion Molecule 1) can also be accumulated in EVs derived from dendritic cells 165, or EVs released from macrophages can transfer their surface antigens to dendritic cells and thus stimulate CD4^+^ T cell activation 166. Moreover, it is possible to modify the surface of EVs via engineering methods, especially genetic engineering. Tian et al. 167 modified the surface of dendritic cell-derived EVs by introducing iRGD peptide-bound pEGFP-C1-RVG-Lamp2b plasmid to deliver doxorubicin to breast cancer cells 168. Chimeric antigen receptor (CAR)-carrying EVs encoding mesothelin-targeted and Myc-tagged scFv have successfully inhibited the growth of MSLN-positive triple-negative breast cancer cells 167. Genetic engineering techniques transfer molecules such as Interleukin 4, indoleamine 2,3-dioxygenase, and FasL into dendritic cells, targeting distinct tumours 164, 169. Modulation of the phospholipid membrane composition or the addition of a targeting antibody on the surface of EVs is another feasible strategy. Chemical crosslinking can be used for membrane engineering. Therefore, immune cell-derived EVs in the peripheral blood might be disease-specific biomarkers of tumourigenesis and inflammation. In addition, immune cell-derived EVs combined with chemotherapeutic agents can prohibit tumour growth.

### Plant exosome-like nanovesicles

Lastly, innately bioactive molecules in plant exosome-like nanovesicles (PELNVs) are suitable, efficient nanocarriers for medical applications. The role of PELNVs in regulating immunological reactions for hemostasis of the gastrointestinal system has been widely studied 170. In cancer therapy, PELNV-derived re-engineered nanovectors target cancer cells as non-immunogenic vehicles 171. For example, ginger-derived ELNVs were successfully used to deliver doxorubicin to colon tumour cells. Interestingly, the antitumour effect was attributed to doxorubicin as well as the inherent nature of this plant-based nanovector that suppresses oxidative stress and the release of inflammatory cytokines. In addition, the researchers proposed that ginger-derived nanovectors increase the release of doxorubicin in the acidic niche of the tumour and thereby diminish its systemic side effects.

**Figure 15:**
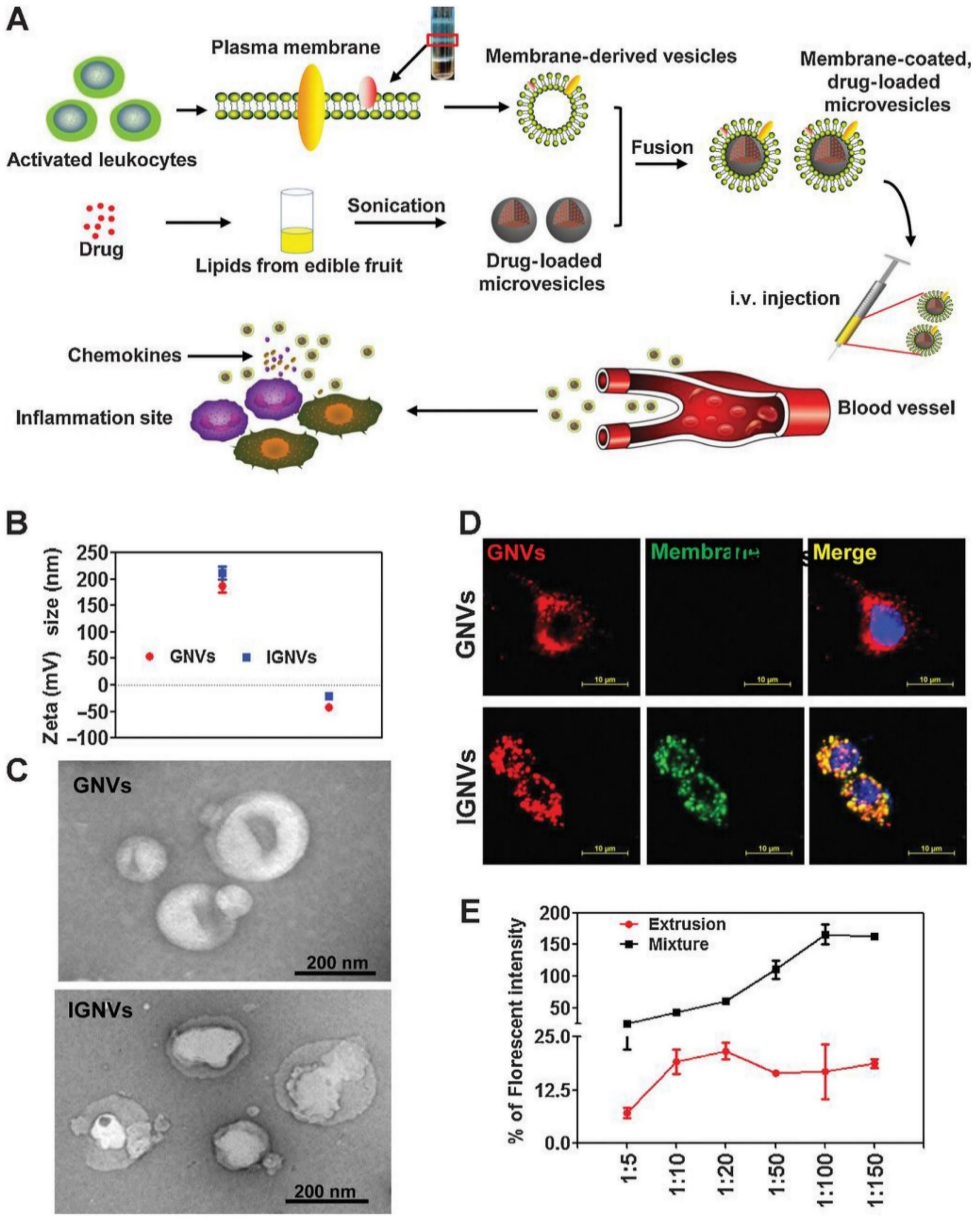
Characterization of plasma membrane-coated grapefruit-derived nanovectors (IGNVs) characterization. (A) Preparation and drug loading steps. (B) Surface zeta potential measurement. (C) Scanning electron microscopy free GNVs (top) and IGNVs (bottom). (D) Co-localisation of the EL4 cell-derived plasma membranes and GNV cores. (E) Fluorescence resonance energy transfer-based measurements of IGNV formation. Reproduced with permission. Copyright Elsevier (2019).

Moreover, studies showed distinct advantages of these nanovectors, such as being biocompatible, cost-effective, and innocuous 172. Grapefruit-derived nanovectors have also been developed for effective and simultaneous delivery of paclitaxel and folate to the SW620 and CT26 colon cancer models in immunodeficient mice. Of note, this nanovector did not traverse the placental barrier in intravenous injection *in vivo* 173. Wang et al. 174 developed a chemokine receptor-enriched plasma membrane-coated grapefruit-derived nanovector, which could efficiently deliver curcumin (an anti-inflammatory agent) and doxorubicin to inflamed CT26 colon tumours and 4T1 breast tumours in mouse models (Figure 15).

The plant source of PELNVs determines the therapeutic effects of these nanovectors. For example, hydrolysis of naringin (the key flavanone of grapefruit) to naringenin by gut microbiota facilitates the antitumour effects of grapefruit-derived nanovectors 175. Furthermore, PELNVs can inhibit the signaling cascades that lead to the expression of proteins involved in tumour development. In this context, PELNVs can disrupt the expression of cyclin D1 mRNA by activating cyclic guanosine monophosphate (cGMP)-dependent protein kinase and thus exert antitumour effects 176. PELNVs also can increase the expression of pro-apoptotic factors and suppress angiogenic molecules 177. Thus, bearing in mind all of these aspects, a combination of natural PELNVs and chemotherapy could serve as a viable plan to eradicate cancer in the future.

Currently, three clinical trials investigate the effects of PELNVs: grape-derived ELNVs for the prevention of oral mucositis in combination with chemoradiation therapy for head and neck cancer (NCT01668849), aloe- and ginger-derived ELNVs for the treatment of polycystic ovary syndrome (PCOS) (NCT03493984), and curcumin-containing PELNVs in the treatment of colon cancer (NCT01294072).

## Influence of size, shape, and surface character of nanovectors for drug delivery applications

Size, shape, and surface chemistry of nanovectors are the key determinants of their drug delivery capacity 178, 179. To achieve a long circulation time, the optimum size range of nanovectors is between 20-200 nm. Nanoparticles smaller than 20 nm are rapidly cleared by the kidney, while nanoparticles larger than 200 nm are easily uptaken by macrophages 180-184. Particles larger than 3 µm in diameter can obstruct microvascular capillaries and also exhibit high macrophagic uptake 184-186.

Moreover, the shape can also influence the delivery properties of nanovectors. Recent studies on particles with different shapes have shown an evident influence of the shape on circulation, lifetime, and macrophage clearance 187. While spherical shapes tend to follow hydrodynamic forces with little lateral movement, non-spherical particles such as rods can undergo some lateral drift 188, 189.

In addition, the nanovector surface structures decide over protein corona formation and cellular uptake mechanism. For example, surface charges or short dense steric surface stabilizers (e.g. poly(ethylene glycol) (PEG)) were reported to increase the mucosal layer and, thereupon, the blood-brain barrier penetration 190, 191.

## Conclusions and Future Outlook

In this review article, a wide variety of auspicious nanovectors for application in multimodal cancer therapy and diagnostics are discussed in terms of mode of action and pharmacokinetics. Promising nanovectors are made of diverse formulations including gold nanostructures, SPIONs, semiconductor quantum dots, mesoporous silica nanoparticles, phospholipid-based liposomes, PAMAM dendrimers, polymeric micelles, extracellular and exome vesicles. Surface functionalization and drug loading of these nanovectors are achieved by conjugation with anticancer drugs, targeted molecules, DNA, siRNA, aptamer, and other functional biomolecules. Depending on the nanovector material and surface chemistry, the drug release inside tumour tissue can be triggered by ultrasound, NIR light pulses, X-rays, or pH value changes. Applications of organic or inorganic nanovectors in cancer therapy include chemotherapy, radiotherapy, immunotherapy, magnetic hyperthermia, and photodynamic therapy, while nanovectors containing gold or SPIONs iron oxide can be in addition used as CT and MRI contrast agents to visualize tumor tissue and metastases.

For future applications in cancer therapy, nanovectors should comprise at least 2 functionalities, attack tumor cells at 2 to 3 targets, and enable targeted cancer therapies. For example, iron oxide and gold nanostructure-based nanovectors should possess surface structures enabling not only binding to anticancer drugs but also conjugation to tumor-cell targeting aptamers, metabolites, or biomolecules to make such treatment most effective. These multifunctional nanovectors may serve first as MRI or CT contrast agents and subsequently be used for tumor tissue visualization. Alternatively, they can be applied for local enhancement of radiotherapy, chemotherapy, hyperthermia, and photodynamic therapy. In the case of nanovectors based on micelles, dendrimers, or vesicles, which are also equipped with tumor-targeting functional groups but encapsulate anticancer drugs, the controlled release of the drugs inside tumor cells plays a particularly important role. Depending on the location of the tumor in the body, the treatment can be triggered with sound waves or NIR laser radiation. Moreover, hyperthermia or photodynamic therapy can be driven by a temperature increase to guarantee a controlled release of the drug payload.

In light of the preceding, there is a myriad of nanomaterials that can redefine the field of cancer treatment in the upcoming future. Given their unique properties, their application as nanovectors seems highly viable, judging by the outcomes of the initial studies described above. The realization of this vision currently hinges upon sufficient demonstration of the lack of toxicity and the development of scalable methods of their synthesis to deploy this concept in cancer theranostics.
